# Editorial: Inclusive leadership in multicultural teams: innovations, challenges and solutions

**DOI:** 10.3389/fpsyg.2026.1963455

**Published:** 2026-09-14

**Authors:** Samyia Safdar, Shaista Noor, Namra Mubarak, Shazia Faiz, Filzah Md Isa

**Affiliations:** 1 Department of Strategy, Leadership & Enterprise, Teesside University International Business School, Middlesbrough, United Kingdom; 2 Cardiff School of Management, Cardiff Metropolitan University, Cardiff, United Kingdom; 3 Department of Management Sciences, Capital University of Science and Technology, Islamabad, Pakistan; 4 School of Business & Law, Taylor's University, Subang Jaya, Malaysia

**Keywords:** cross-cultural leadership, cultural intelligence (CQ), employee wellbeing (EWB), inclusive leadership (IL), multicultural teams diversity and inclusion, organizational psychology, team innovation

In leadership and organizational psychology studies, the development of inclusive leadership qualities has become increasingly important due to the rapidly growing multicultural workforce in modern workplace (Agba and Agba, 2025). The interaction between individuals from diverse professional, social, and cultural background has been intensified due to workplace mobility, increasing globalization, and hybrid and remote workplace arrangements. Although these advances create opportunities for greater creativity and innovation, they also present challenges related to equitable participation, social cohesion, psychological safety, and effective communication. Therefore, exploring how leaders can effectively manage diversity and promote inclusion has become an increasingly crucial area of focus for practitioners and researchers. Inclusive leadership has been largely conceptualized as a leadership approach characterized by fairness, accessibility, openness, and appreciation of individuals' distinctiveness, while simultaneously fostering a sense of belonging among group members (Randel et al., 2018). Numerous studies have demonstrated that inclusive leadership positively impacts employee engagement, innovation, wellbeing, and organizational performance (Bao et al., 2022; Wang et al., 2021; Umrani et al., 2024; Gong et al., 2021). Yet, significant gaps persist in our understanding of how inclusive leadership works within multicultural settings. Especially, related to the contextual factors shaping leadership effectiveness, the psychological mechanisms underlying its effects, and the challenges arising by evolving organizational forms and emerging technologies. These challenges are particularly significant in multicultural groups, where variations in values, social norms, identities, and communication styles may complicate team relationships and interaction. Additionally, the rapid adoption of artificial intelligence and digital technologies has created new opportunities for enhancing inclusion while simultaneously raising concerns regarding equity, bias, and the preservation of human-centered leadership practices. Addressing these concerns requires a multidisciplinary perspective that integrates organizational psychology, leadership theory, technological innovation, and cross-cultural research. The Research Topic “*Inclusive leadership in multicultural teams: innovations, challenges and solutions*” was designed to advance understanding of these complex dynamics. Specifically, it aimed to explore how inclusive leadership shapes individual and team outcomes, identify the social and psychological mechanisms underlying inclusion, examine the risks and opportunities associated with technological advancements, and identify evidence-based approaches for promoting inclusive and high-performing multicultural workplaces. Four major themes were identified from the 14 articles published within this Research Topic, each contributing meaningfully to its overall objectives. These themes include: (1) inclusive climates, employee voice, and identity; (2) leadership capabilities and psychological mechanisms; (3) diversity, institutional inclusion, and technology; and (4) creativity, innovation, and team dynamics. The first thematic area focuses on inclusive climates, employee voice and identity. It has been identified that inclusive leadership influences the employee outcomes through central mechanisms namely belongingness and identification (Li et al., 2025). Öz and Meriç found that teachers' organizational citizenship behaviors, including sportsmanship, civic virtue, conscientiousness, and altruism, increase when they identify strongly with their organization. This highlights the significance of identification as a predictor of positive employee outcomes. Similarly, Shen and Shen found that employee voice behavior is encouraged in the organizations that promote an inclusive climate. Their results confirms that organizations with inclusive climates enhance employees' confidence in communicating ideas by enhanced voice efficacy. Additionally, satisfaction with voice channel construct strengthens this relationship, especially when formal voice systems are perceived as accessible and effective. These findings highlight that inclusion requires both institutional structures that enable meaningful participation and supportive organizational climates. Fu et al. study also placed identity processes at center of their study. They found that the relationship between transformational leadership and innovation is partially mediated by organizational identification. This research enhances the understanding of how shared identities can facilitate alignment and collaboration within complex organizational settings. Song et al. explored that inclusion may results in unintentional outcomes. They found that employees who are being trusted by the leaders may become victim of coworker envy which leads toward workplace exclusion. Additionally, these negative consequences mostly occur in highly competitive environments. Therefore, this study points out that a careful execution of inclusive leadership practices is required to avoid unnecessary social tensions.

The second theme area centers on the leadership capabilities and psychological mechanisms. As, it advances inclusion and employee thriving. Gao et al. investigates how new generation employees thrive at work, and their job performance is being affected by intellectual humility leadership. Based on social exchange theory and job demand-resources theory, they concluded that intellectual humility leadership which advances positive job attitudes, consequently, enhances subordinate performance and thriving at work. They further highlighted that these effects are stronger among subordinates with higher core self-evaluations, emphasizing the interaction between individual characteristics and leadership behaviors. Correspondingly, Xing et al. explored how leader's emotional intelligence enhances team innovation. They examined how underlying mechanism namely knowledge sharing and psychological safety that influence this relationship. Their findings showed that leaders with high emotional intelligence who create psychologically safe environments contribute to greater team innovation. This highlights the significance of the relational and affective dimensions of leadership within culturally diverse teams.

Liu X. et al. also investigated the leadership effectiveness by exploring the impact of team leadership on the professional development of university teachers. They concluded that organizational innovation climates strengthen the effects of leadership across the proposed pathways, while innovative self-efficacy partially supported the moderated mediation model tested in their study. These findings highlight the importance of supportive organizational contexts in enhancing the benefits of inclusive leadership. Accompanying these conclusions, Tian et al. investigated how leaders' egoistic and altruistic values influence employee happiness and leadership effectiveness. Their findings indicate that altruistic values enhance employee wellbeing through cognitive and emotional pathways, whereas egoistic values do not generate the same level of benefits. These findings suggest that value-based leadership plays a crucial role in fostering collaborative and supportive work environments.

The third theme focuses on diversity, institutional inclusion and technology in current organizations. Javeed et al. examined the relationship between diversity and resilience, by exploring the role of gender diversity in marketing and financial decision during organizational disasters. They concluded that gender based diverse leadership groups show more empathetic communication and greater brand resilience. Guo et al. further explored the role of Chinese trade union in advancing employment amongst migrant workers. They found that the role of union is directly linked with employment outcomes. The union participation results in positive employment outcome by enhanced social insurance coverage, formal contracts, and labor protection. Lastly, Tahir et al. investigated the emerging role of artificial intelligence in inclusive leadership within culturally diverse educational teams. Their theoretical investigation proposed a governance framework highlighting transparent AI protocols, interdisciplinary governance structures, human oversight, and equity audits. The study highlighted that inclusive and ethical AI implementation is necessary to promote fair leadership practices and prevent the reinforcement of existing gender inequalities.

The final theme centers on creativity, innovation, and team dynamics. It is widely recognized that inclusive leadership regarded innovation. However, the mechanisms through which the leadership influences creativity remain an emerging area of research. Qiao et al. explored the relationship among transformation leadership and team innovative performance. They concluded that leaders could foster persistent psychological sources like patient capital, and that backing innovation. Zhao and Ren explore the role of organizational system in enhancing creativity. They found that diverse and inclusive human resource practices (DIHRP) improve employee creativity. They concluded that supplementary and complementary person organization fit boost the impact of DIHRP on creativity, while inclusive leadership play the role of accelerator between the direct and indirect relationship of DIHRP and creativity. This study demonstrates how HR systems and leadership work together to enhance creativity at diverse organizational settings. In contrast, Liu Y. et al. focused on the harmful effects of non-inclusive leadership via their study of team variance surroundings. They concluded that differentiating between insider and outsider can reduce the team creativity due to diminished joint decision-making and collaboration. They further argued that team empathy plays a significant role in mitigating these negative effects. Overall, their findings highlight the importance of fostering inclusive team climates and emotional support to sustain workplace innovation.

Collectively, the studies included in this Research Topic provide a broad and multifaceted understanding of inclusive leadership in culturally diverse teams. The findings reveal that inclusive leadership extends beyond interpersonal behaviors to encompass technological systems, identity processes, institutional structures, and organizational climates. These studies further demonstrate that inclusion is not merely a moral obligation but also a strategic resource that enhances creativity, innovation, resilience, wellbeing, and organizational effectiveness. In today's rapidly changing world, organizations continue to evolve in response to demographic shifts, globalization, and technological advancements. Consequently, managing these complex organizational environments through inclusive leadership will remain an important area of investigation. The findings presented in this volume provide valuable practical guidance and theoretical insights for practitioners, researchers, and policymakers seeking to build innovative, inclusive, and sustainable organizations capable of thriving in increasingly complex and diverse environments.
